# 

**Published:** 1886-02

**Authors:** 


### CONTENTS.

ORIGINAL ARTICLES.— Poisoning by Coal Gas of Two Comanche Chiefs — Ante Partum Hæmorrhage— Dr. Otto Ringk's Theory and Treatment of Diphtheria—A Case of Uræmia Complicating Labor... 343–359
CULLINGS FROM CONTEMPORARIES. — Large Fæcal Abscess, Spontaneous Closure, etc—A Medical Iconoclast .......... 361-362
ABSTRACTS FROM FOREIGN EXCHANGES .......... 363
CORRESPONDENCE.—Our New York Letter .......... 366
SOCIETY NOTES.—Williamson County Medical Society— Washington County Medical Society — Cameron County Medical Society—Circular, "Texas Quackery"—Next Meeting Texas State Medical Association—Galveston Medical Club .......... 369–373
EDITORIAL.—Death of a Distinguished Texan, Dr. Ashbel Smith—A Lordly Fraud in Texas—Our Say on the Subject. .......... 376–380
EDITORIAL NOTES.—The Late Dr. Ashbel Smith—Announcement—A Journalistic Elsler—Piracy and Obstinacy — Extraordinary Announcement for 1886—The Florida Medical and Surgical Journal—A Disgraceful Act—Bureau of Health .... 382–386
BIBLIOGRAPAY. – Book Notices and Reviews — Books Received .......... 386
ADVERTISERS' NOTICES .......... 389

